# Experimental analysis on impact load of flash flood against a passable structure

**DOI:** 10.1371/journal.pone.0287848

**Published:** 2023-08-07

**Authors:** Sen Wang, Hanwu Zheng, Er Huang, Xingnian Liu, Ming Luo

**Affiliations:** State Key Lab of Hydraulics and Mountain River Engineering, Sichuan University, Chengdu, 610065, P. R. China; University of Limpopo, SOUTH AFRICA

## Abstract

Flash flood in mountainous regions have caused significant damage worldwide in recent years, impacting and destroying structures. The impact load of flash flood is the key factor in the process of destruction. In this study five existing models for impact pressure calculation were compared and analyzed based on experimental data. What’s important, combining two existing models, a new model considering both hydrodynamic and hydrostatic pressure was proposed. The results showed that the relative error of empirical coefficient was lowered from 16.2% to 7.09% in the condition of Froude number less than 2, and lowered from 10.7% to 9.9% when the Froude number exceeded 2 in the new calculating model. Besides, the distribution of fluid impact and the maximum pressure point against a passable obstacle was discussed. The findings of this research could provide ideas for structure design to withstand the flooding impact.

## 1 Introduction

Flash flood in mountainous regions have caused significant damage worldwide in recent years [1,2]. When flowing through the residence community, flash floods hit and destroy the structures severely and cause a significant impact on people’s lives. The impacts of flash floods can be divided into two parts: fluid impact and solid impact [3]. Due to the larger density of solid material, solid impact has several hundred times more energy than the fluid impact and is always more threatening. However, as fluid is the driving force of solid material and can also generate more stable impacts, many researchers have solely focused on fluid impact [4–9].

Field experiments and observations are the most direct and persuasive way to analyze the physical mechanisms hidden in flood disasters [10–13]. However, due to limitations in the field, such as difficulties in installing measurement devices and the complex nature of fluids, collecting data through this method has not been convenient until now. To overcome these limitations, simplifications are necessary for conducting flume experiments. Thus, abundant works have been conducted through flume experiments to analyze the fluid impact [4,6,7,14–16]. In terms of fluid, water depth and flow velocity are considered the important parameters. To physically evaluate the impact force of fluids from flow characteristics, flow pressure has been introduced and divided into two methods based on physical significance: hydrodynamic pressure by 1/2*ρV*^2^ and hydrostatic pressure by *ρgh*

In the hydrodynamic pressure method, the fluid impact is determined by the product of flow velocity and unit water weight, and an empirical coefficient is used to represent amplifying effects, which can be calibrated using experimental data [9,10,17–20]. The conversion of energy is also considered in this method, that is, when the fluid flow encounters an obstacle, the flow incident velocity theoretically decreases to zero and gradually transforms into pressure against the obstacle. Cui et al [9] proposed an experimental power hydrodynamics model using the Froude number and flow velocity to calculate the hydrodynamic pressure of the slurry based on datasets from miniaturized flume experiments and field events, which yielded good results. Canelli et al [17] conducted a flume experiment specifically designed to analyze the dynamics of debris flow impact on various structures. They developed an analytical model to estimate the thrust induced by debris flow on any structure. Hübl and Holzinger [18] developed a hydrodynamic pressure formula by using a regression analysis between the normalized impact pressure and the Froude number, also showing a good result. Zhang [10] and Armanini [21] conducted flume experiments and considered several empirical coefficient values which was related to the uniformity of fluid and bed slope. Watanabe [22] presented a coefficient value for laminar flow and fine-grained material, while Eglit et al. [23] suggested a higher value up to 4.0 for coarse material. However, this method is a simplistic and rapid approach that disregards certain internal flow mechanisms, such as flow patterns and turbulence. Flow patterns, which are divided into supercritical and subcritical flow, significantly affect the impact against the obstacle. Therefore, some researchers have attempted to explore the relationship between the empirical coefficient and the Froude number which was utilized to describe flow patterns [9,14,18]. Meanwhile, the Reynolds number was considered the variable of empirical coefficient based on flow turbulence, and a fitting curve was proposed in the debris field [24].

In the hydrostatic pressure method, it’s assumed that *ρgh* is the main factor in the controlling equation and an empirical coefficient is also essential. Several examples of the hydrostatic model were given by Lichtenhahn [25], Aulitzky [26], Armanini [21], and Armanini et al [4]. In the formula proposed by Lichtenhahn [25], the only free parameter was the height of debris flow. And it was often equal to the height of the barrier structures. Armanini [21] assumed that the hydrodynamic force exerted by a debris flow on a fixed structure was proportional to the hydrostatic pressure on the structure based on some common practice, which was supported by experimental evidence on the collapse of some check dams [26]. Armanini et al [4] assumed that the distribution of the fluid pressure against the surface of the obstacle was hydrostatic, and the maximum height of the fluid on the surface of the obstacle was obtained by using the energy equation and momentum equation for different flow patterns.

Currently the hydrodynamic model proposed by Hübl and Holzinger [18] and the hydrostatic model proposed by Armanini [21] has been identified as possible design equations to deal with debris flow barriers for engineers. In addition to these two main methods, there are solid body collision models based on Newton’s impact theory and elastic models for considering the impacts of single boulders within the debris flow. Examples of such models can be found in the works of Yu and Tuan [27], Chen and Tang [28], and He et al. [29]. Chen and Tang [28] developed a new method to calculate the impact force of debris flow using the second Newton’s law by calculating velocities and acceleration of every phase, and the error of the model was relatively low. Also, based on the Hertz contact theory, He et al [29] presented a theoretical method to calculate the impact force of outrunner block in a debris flow on structures, and the method matched well with practice. Besides, impact models using shock wave theory by compressible flows were presented by Eglit et al [23]. In the paper, estimations of the impact pressure were based on the scheme of shock waves, and the author further developed the earlier theory to account for thermodynamic equations. However, as some required particular input is difficult to obtain for real world debris flows, these above models are difficult to apply. Therefore, this paper will focus on hydraulic-based methods.

In general, the hydrodynamic pressure by 1/2*ρv*^2^ and the hydrostatic pressure by *ρgh* are commonly used to calculate the total pressure. However, two main issues need to be addressed. First, different values of the empirical coefficient are used in various research studies. Second, there is a lack of analysis on the distribution of the fluid impact. Therefore, this research aims to (1) compare different models and the empirical coefficients (2) propose a new pressure calculating model based on our findings (3) discuss the distribution of fluid impact pressure against an obstacle and determine the maximum pressure point.

## 2 Experimental method

### 2.1 Experimental equipment

**Fig 1:**
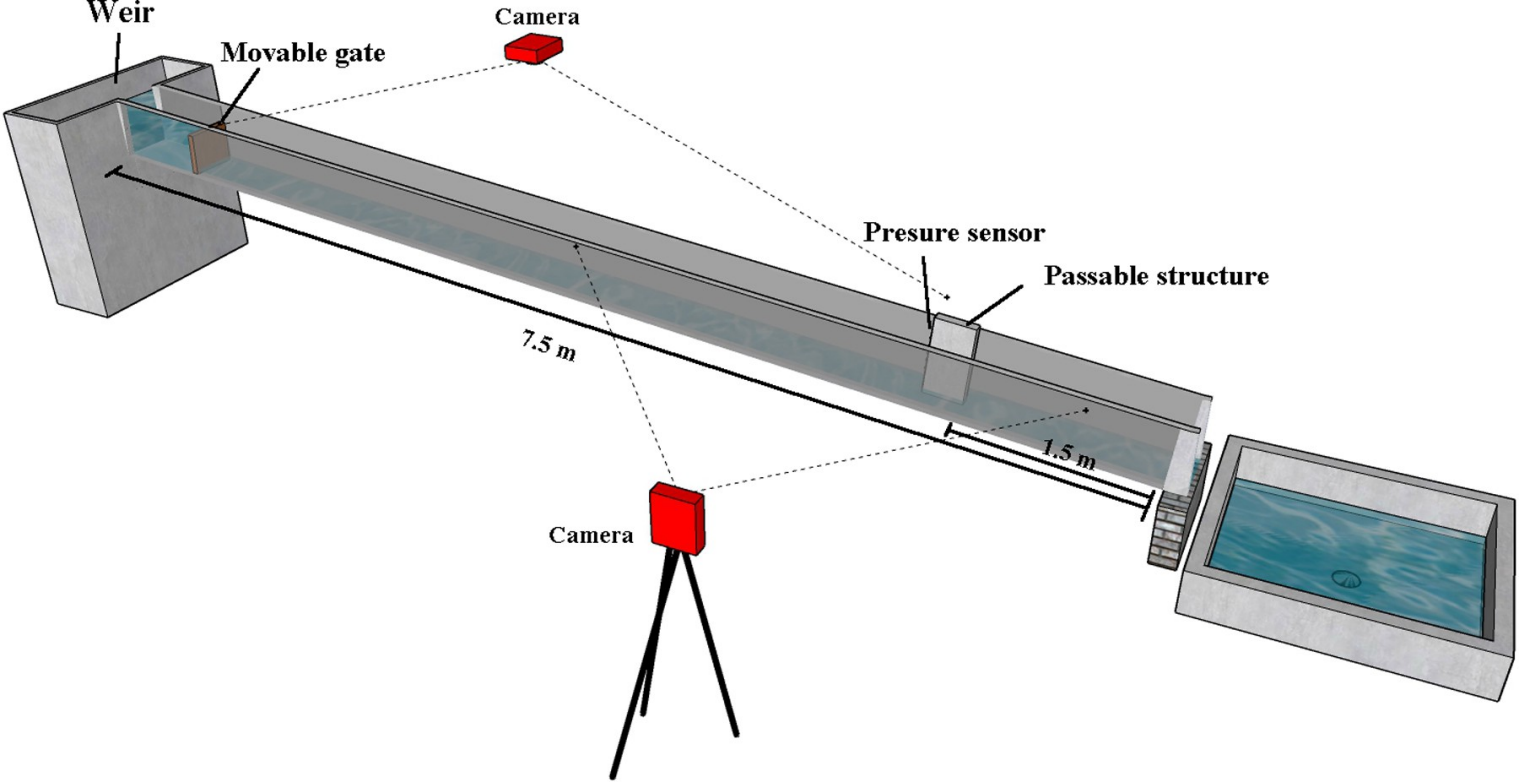
Schematic of flume experiments.

The experiment was conducted in the State Key Laboratory of Hydraulics and Mountain River Engineering, Sichuan University. The flume is 7.5 m long, 0.4 m wide, 0.4 m deep and it is made of transparent glass to allow for optical observation of the experiment process (Fig 1). Besides, some transparent square paper (2mm×2mm) is pasted on the lateral side of the flume for observation of water depth. The flow is regulated by a rectangular weir with a wide top at the inlet, generating a steady uniform flow in the open channel. Not far downstream the weir, there is a movable gate stuck in a slot to guarantee a required water level. In the flume an obstacle is fixed 1.5 m upstream of the outlet and the shape of the obstacle is prismatic, with dimensions of 0.25 m in length, 0.06 m in width, and 0.5 m in height. The outlet of the flume is free, and the slope could be altered through adjusting the height of the outlet in the experiment.

**Fig 2:**
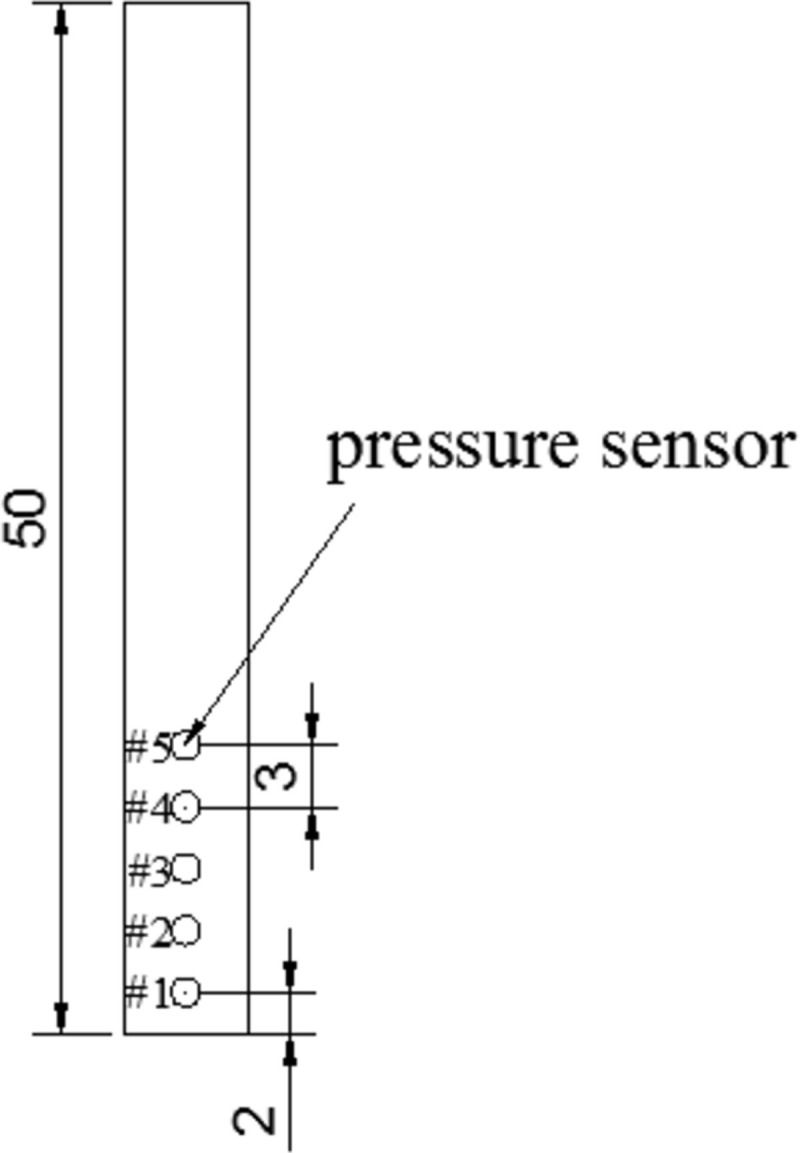
Pressure sensor arrangement. (All schemes are quoted in cm).

Flow velocity and water depth of the incident flow were measured to detect the impact pressure of the flash floods against an obstacle. For this purpose, two cameras were used in this experiment. One camera was fixed above the flume to record the flash floods’ average surface velocity, while another camera was placed on the lateral side of the flume to measure the water depth of the incident flow, and these two were located 4.0m, 3.0m upstream of the obstacle, respectively. What’s important, to record the impact pressure of the flash floods against the obstacle, five pressure sensors (CYYZ31 produced by Star sensor manufacturing Co., Ltd) were attached to the upstream surface of the obstacle uniformly along a vertical direction, with sensors positioned near the root of the impact area upon the obstacle. The measuring range of pressure sensors is 10kPa, and accuracy level is 1% which matched well in this experiment. The arrangement of pressure sensors is shown in Fig 2. The sensor #1 is located 2cm from the bottom of the obstacle, and the distance between any two sensors is 3cm.

### 2.2 Experiment processing

To simulate the impact of a flash flood against an obstacle, water was stored in the upstream weir and a gate was set in the downstream for generating a flash flood with sufficient magnitude. Meanwhile, slopes of 0.5%, 1%, 2%, 4% and 6% were used to study the impact characteristics over a wide range of Froude numbers (S1 Table). The experiment began when water in the upstream reached a corresponding level and the outgate opened. Then water entered the flume and hit the obstacle. After a short pause, the water surface was stable, then polystyrene spheres were released upstream of the flume to record the flow surface velocity. When these spheres flowed across all the tested regions, one test was completed.

The velocity was calculated by the average value of filtered spheres during each test. Initially, a total of 200 spheres were used to evaluate the average velocity, but some filters were required. The reasons were as follows: first, on low slopes of 1% and 0.5%, flow in the upstream was susceptible to the backwater effect. During the experiment there was a period of disturbance in water surface in front of the passable obstacle due to the blocking effect, hence the water depth here was not stable enough and the velocity in front of the passable obstacle could not be obtained. Second, on high slopes of 2%, 4% and 6%, the impact pressure may be cut down when water depth of the incident flow was lower than the bottom pressure sensor. Meanwhile, the water depth was obtained from the corresponding camera frame, and if the decline during the adjacent time interval was slow, the point value was acceptable. In the experiment the pressure of the fluid against the obstacle oscillated during the impact, especially on high slopes such as 2%,4%, and 6%, the pressure with a sampling interval of 1s was selected.

As mentioned above, the velocity in front of the passable obstacle could not be obtained immediately on low slopes of 1% and 0.5%, so the data should be transformed in these two conditions. The water depth and flow velocity in the upstream were recorded, as with water depth in front of the passable obstacle. Therefore, the energy equation could be used to calculate the velocity in front of the obstacle, as follows:

$$h_{0}+\frac{V_{0}^{2}}{2g}=h_{1}+\frac{V_{1}^{2}}{2g}+ids$$ (1)

where *h*_0_ (m) and *V*_0_ (m/s) are the water depth and flow velocity in the upstream respectively; *h*_1_ (m) and *V*_1_ (m/s) indicate the water depth and flow velocity of the flow in front of the obstacle, respectively; *i* is the slope; *ds* (m) is the distance between two cross-sections; *g* is the acceleration of gravity (9.8m/s^2^).

In this study a total of 88 tests were conducted with varying flow velocities and water depths by filtering out unqualified data and transforming some of it. Based on the experimental data, the Reynolds number was estimated in a range of 2.0×10^4^ to 1.2×10^5^ and the Froude number ranged from 1 to 6.5(S1 Table). The two dimensionless numbers were calculated using Eq (2) and Eq (3) respectively, as follows:

$$Re=\frac{VR}{v}$$ (2)

$$Fr=\frac{V}{\sqrt{gh}}$$ (3)

in which *V* (m/s) is the flow velocity; *R*(m) is the hydraulic radius; *v* is the kinematic viscosity and the value is set to be 1×10^-6^ m^2^/s; *h* (m) is the water depth.

## 3 Results and discussion

### 3.1 Verification and comparison of different calculating models and the improvement

**Fig 3:**
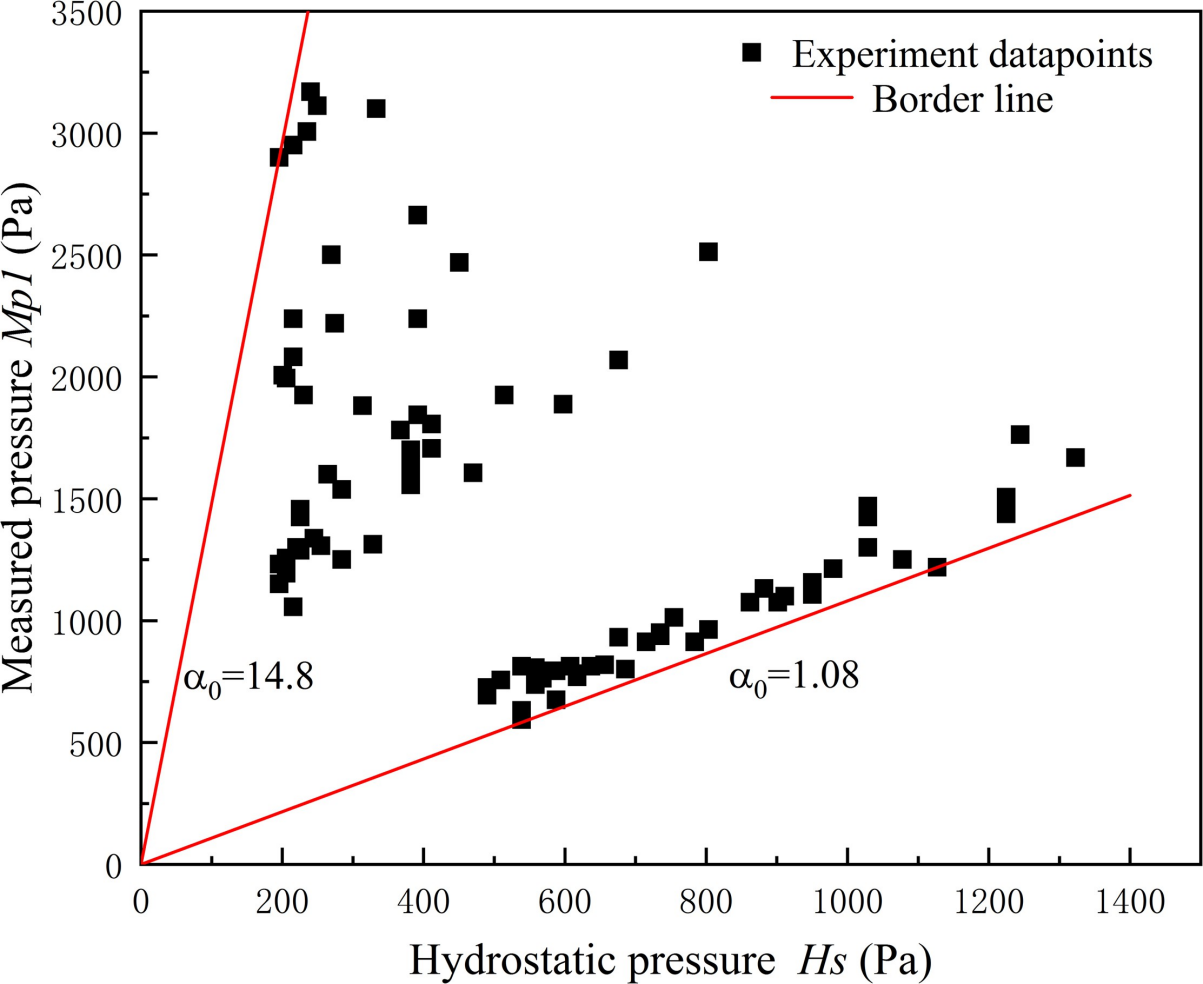
The relationship between measured pressure *Mp1* and hydrostatic pressure *Hs*.

To compare different models and the empirical coefficients, the data from sensor #1 was used in this part. As different methods are available to estimate the fluid impact based on the characteristics of the incident flow, one of them calculating the impact pressure is as follows,

$$P=\alpha_{0}\rho gh$$ (4)

where *α*_*0*_ is an empirical coefficient, *ρ* is the density. In this study *α*_*0*_ was calibrated using experimental data as Fig 3 showed. It indicated there was no single line that could fit all scatters, which meant the calculated pressure cannot be related to the measured pressure considering only one constant. Also, a range of value was defined in some research. Lichtenahahn [25] inferred 7 to 11 of *α*_*0*_ and Scottonand and Deganutti [30] suggested a range of 2.5 to 7.5. Based on our experimental data, the range was 1.08 to 14.80.

The hydrodynamic pressure was not taken into account in Eq (4). However, it always occupies a large part of the total pressure in high velocity conditions. Therefore, this model was uncomprehensive and the range of *α*_*0*_ was unpredictable when the flow velocity varied widely. Eq (4) is more appropriate for slower flow velocities, whereas higher velocities require a consideration of the kinematic component.

**Fig 4:**
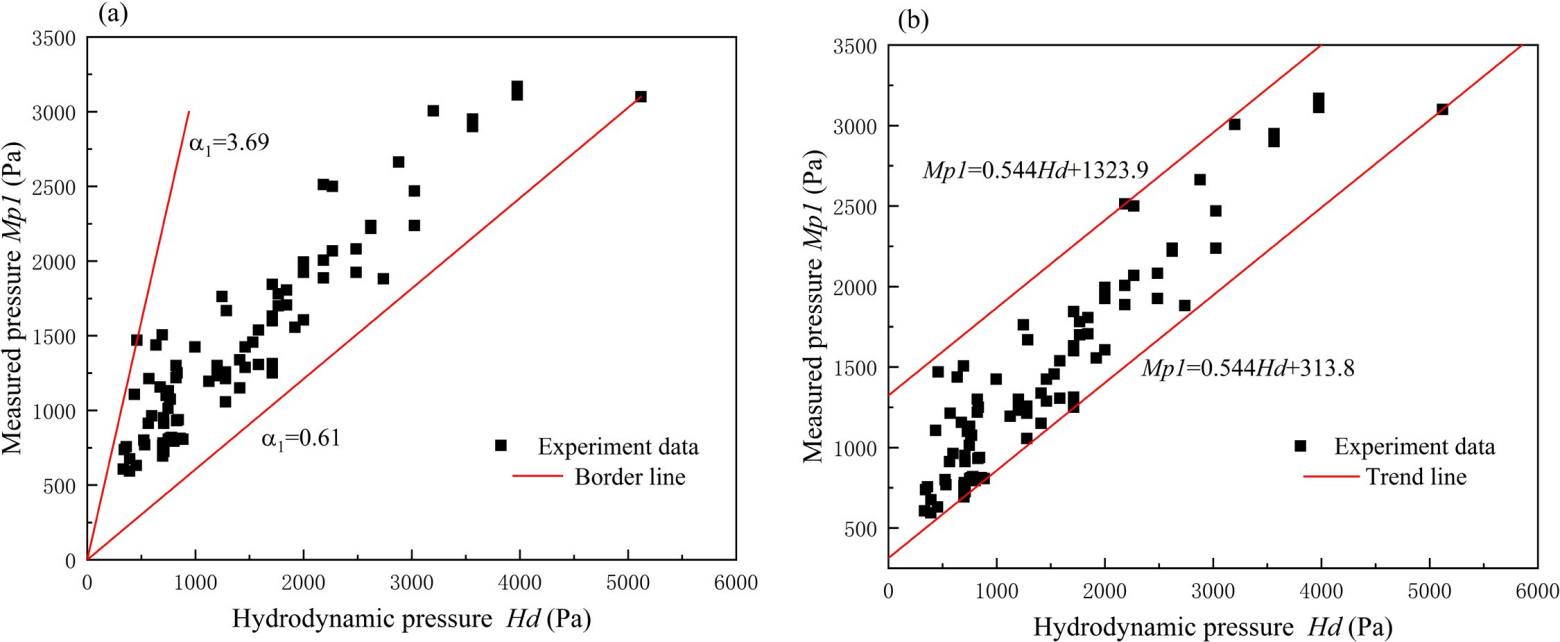
The relationship between measured pressure *Mp1* and hydrodynamic pressure *Hd*. (a) border line; (b) trend line.

There existed another model calculating the total pressure as follows:

$$P=\alpha_{1}\cdot \frac{1}{2}\rho V^{2}$$ (5)

where *α*_*1*_ is a constant coefficient. The medium part for estimating pressure in Eqs (4) and (5) was different. As the velocity is always high, it is more destructive when debris flows and floods occur in mountainous areas. In this condition, the kinematic pressure makes up the main part of the actual pressure and has also been studied as a crucial factor by many researchers. Zhang [10] conducted a field experiment in Jiangjia Ravine, China. In the experiment, the flow was considered to be a viscous debris and a range of 3 to 5 of the empirical coefficient *α*_*1*_ was calibrated. Armanini and Scotton [31] selected a PVC material and water mixture to conduct the granular flow, and a range of 0.45 to 2.2 was calibrated of the coefficient *α*_*1*_. In our study, based on the experiment data, the relationship between measured pressure and the hydrodynamic pressure calculated by 1/2*ρV*^2^ was illustrated in Fig 4A. It indicated the two were with positively correlation. In some cases, the calculated pressure which ignored the hydrostatic pressure was larger than the measured pressure, this may be due to more energy losses and non-uniform velocity distribution when the fluid flowed against an obstacle in the experiment. From the calibration of scatters, the range of *α*_*1*_ was 0.61 to 3.69. What’s important, an improvement can be made to the model expressed by Eq (5). That is if we use a trend line to depict the tendency of the scatter (Fig 4B), a constant term should be added on the right-hand side of Eq (5), as follows:

$$P=\alpha_{2}\frac{1}{2}\rho V^{2}+c$$ (6)

in which *α*_*2*_ is an empirical coefficient and the constant *c* ranged from 313.8 to 1323.9 in our experiment. When the flow velocity was 0, 1/2*ρV*^2^ equaled to 0 and *P* equaled to *c*. Hence *c* represented the hydrostatic pressure and its range was almost within the value range of the hydrostatic pressure by *ρgh*. However, it is worth noting that the scatters may be inadequate in conditions of high measured pressure. These conditions would be captured when the hydrostatic pressure by *ρgh* increased to a large value, resulting in the scatters more similar to an area than a line. On the other hand, in conditions of low measured pressure, the scatter cannot extend indefinitely because the energy loss cannot increase at the same scale as the velocity. This model is an improved version of Eq (5) and can extract the influence of the hydrostatic pressure from *α*_*1*_, thereby providing a clearer understanding of the mechanisms of fluid impact.

When considering velocity and water depth of the incident flow as important factors for the total pressure, the Froude number is often used and the measured pressure of different Froude numbers is examined in some research. Hübl and Holzinger [18], and Cui et al [9] have used a form similar to Eq (5), with an empirical coefficient that is not constant, but a power function related to the Froude number as follows:

$$\left\{\begin{array}{c} P=\alpha_{3}\frac{1}{2}\rho V^{2}\\ \alpha_{3}=aFr^{b} \end{array}\right.$$ (7)

where *a* and *b* are constant coefficients; *α*_*3*_ is an empirical coefficient. The Froude number is the ratio of flow velocity to the square root of water depth times gravity acceleration. It can also be expressed as a ratio of hydrodynamic pressure to hydrostatic pressure, as follows:

$$Fr=\frac{V}{\sqrt{gh}}=\sqrt{2}\sqrt{\frac{\frac{1}{2}\rho V^{2}}{\rho gh}}$$ (8)

**Fig 5:**
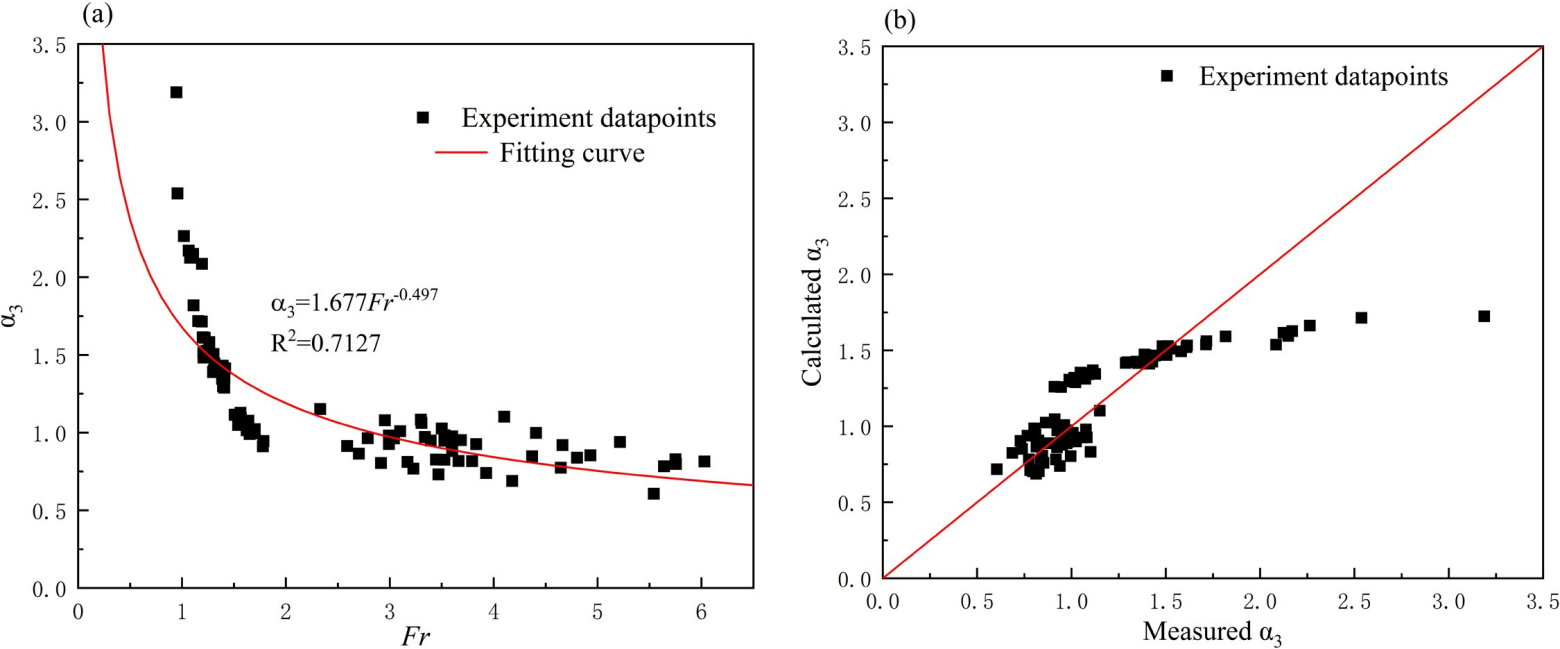
(a) The relationship between *α*_*3*_ and *Fr*; (b) The relationship between calculated *α*_*3*_ and measured *α*_*3*_.

Thus, the physical meaning of the Froude number is clear, and its value is a weight factor of different kinds of pressure. The relationship between *α*_*3*_ (ratio of the measured pressure to the hydrodynamic pressure) and *Fr* was illustrated by experiment data (Fig 5A). A power function relation was obtained based on the scatters group, and the values of calibrated constants *a* and *b* were 1.677 and -0.497 respectively. The relationship indicated that *α*_*3*_ and the Froude number were negatively correlated. As the Froude number decreased, the hydrodynamic pressure by 1/2*ρV*^2^ decreased, leading to an increase in *α*_*3*_ until it approached infinity when the Froude number approached 0. Besides, the correlation between the experiment scatters and fitting curve showed poor performance in the high region of *α*_*3*_ (corresponding to low Froude number), while a well-matched performance was observed in the low region of *α*_*3*_ (corresponding to high Froude number), and the dividing point could be defined as *Fr* = 2. Fig 5B showed the comparison between the calculated *α*_*3*_ using Eq (7) and the measured *α*_*3*_ (ratio of the measured pressure to the hydrodynamic pressure). What’s more, the result in S2 Table indicated the relative error of calculated *α*_*3*_ was between 0.3% and 24.5%, with an average value of 10.7% when the Froude number greater than 2. On the other hand, when the Froude number below 2, the relative error of calculated *α*_*3*_ was between 0.1% and 45.9%, with an average value of 16.2%.

**Fig 6:**
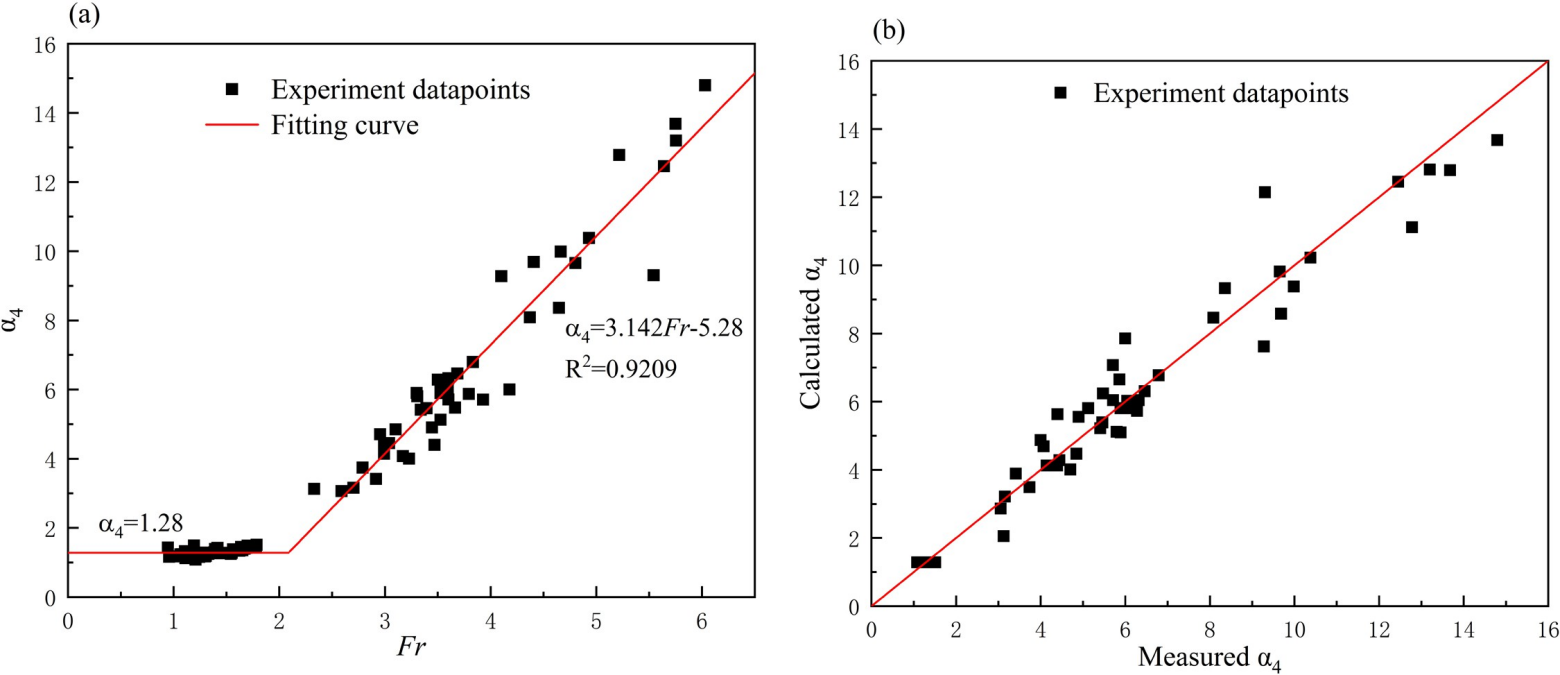
(a) The relationship between *α*_*4*_ and *Fr*; (b) The relationship between calculated *α*_*4*_ and measured *α*_*4*_.

Hydrodynamic pressure by 1/2*ρV*^2^ was used to calculate the total pressure in Eq (7). It is also possible to substitute it with *ρgh*, as they have the same dimensions, as follows:

$$P=\alpha_{4}\rho gh$$ (9)

in which *α*_*4*_ is an empirical coefficient. The relationship between *α*_*4*_ (ratio of the measured pressure to the hydrostatic pressure) and *Fr* can also be illustrated by the experiment data (Fig 6A). The plot can be divided into two parts based on the Froude number values. When the Froude number was below 2, *α*_*4*_ remained almost constant while the scatters fluctuated between 1 and 1.5, with an average value of 1.28. In this section, hydrostatic pressure by *ρgh* accounted for a large portion of the total pressure, while the influence of hydrodynamic pressure by 1/2*ρV*^2^ and other energy losses just accounted for only a small portion. Therefore, *P* would approach to *ρgh*. Meanwhile, if the Froude number exceeded 2, *α*_*4*_ increased as the Froude number increased, and the correlation followed a linear relationship. In this section, hydrodynamic pressure gradually became the main component of the total pressure. And as the proportion of hydrodynamic pressure increased, the measured pressure became many times the hydrostatic pressure, resulting in an increase in *α*_*4*_. The scatters of these two sections can be expressed separately as follows:

$$\left\{\begin{array}{c} \alpha_{4}=3.142Fr-5.28\;\;\;\;\;\;\;Fr>2\\ \alpha_{4}=1.28\;\;\;\;\;\;\;\;\;\;\;\;\;\;\;\;\;\;\;\;\;\;\;\;\;\;\;\;Fr\leq 2 \end{array}\right.$$ (10)

The comparison between the calculated *α*_*4*_ using Eq (10) and the measured *α*_*4*_ (ratio of the measured pressure to the hydrostatic pressure) was shown in Fig 6B. Based on the calculation result (S3 Table), it showed when the Froude number below 2 the relative error of calculated *α*_*4*_ was between 0.2% and 18%, with an average relative error of 7.09%. On the other hand, the range of relative error of calculated *α*_*4*_ was from 0.03% to 34.4% with the average value of 9.9% when the Froude number exceeded 2. Hence the representativeness of calculated *α*_*4*_ was better when Froude number less than 2.

Based on an extensive amount of experimental data, Proske et al [32] verified Eq (9) and suggested a linear relationship between *α*_*4*_ and *Fr*. However, there was a significant amount of scatter in the data due to differences in experimental designs among researchers. Eq (9) is similar to Eq (7), with both equations having empirical coefficients that are related to the Froude number. The main difference between the two equations is that Eq (7) relates the measured pressure to the hydrodynamic pressure by 1/2*ρV*^2^, while Eq (9) relates it to the hydrostatic pressure by *ρgh*. Additionally, the relationship between the empirical coefficient and the Froude number is different for each equation. Eq (7) is suitable for conditions in which the hydrodynamic pressure is the main contributor of the total pressure, and the hydrostatic pressure is related to the hydrodynamic pressure by *α*_*3*_ while calculating the total pressure. The advantage of Eq (7) lies that *α*_*3*_ is almost a constant when Froude number exceeds 2, then calibration would be easier. What’s more, the same applies to Eq (9) when the Froude number less than 2. Thus, combining the advantages of both equations, a new model is proposed as below:

$$\left\{\begin{array}{c} P=\alpha_{3}\rho V^{2}\;\;\;\;\;\;Fr>2\\ P=\alpha_{4}\rho gh\;\;\;\;\;Fr\leq 2 \end{array}\right.$$ (11)

The model of Eq (11) provides a more convenient and accurate approach compared to the single Eq (7) or Eq (9), as it accounts for both the complicated relationships and provides a more accurate empirical coefficient with low relative error in the respective interval. Additionally, it incorporates both the hydrodynamic pressure by 1/2*ρV*^2^ and the hydrostatic pressure by *ρgh*, which is a great improvement. Therefore, the impact pressure of the fluid against an obstacle can be defined by means of the interval of the empirical coefficient.

### 3.2 The expectation of the distribution of fluid impact pressure

The maximum pressure of the fluid against a passable obstacle along the water depth should be focused when considering the shear strength of the material of the obstacle. As the water depth and velocity of the incident flow increase, the resultant force also increases, and the action point moves gradually away from the bottom of the obstacle, resulting in an increased bending moment in the bottom. This can trigger damage to the obstacle when the bending moment exceeds the resistance moment. However, there is few research on the distribution of fluid impact pressure against an obstacle, making the maximum value and the action point unknown.

In this section, the pressure data from the two bottom sensors (sensor #1 and #2) were utilized to investigate the distribution of flood impact pressure. The point velocity along vertical direction is different, sensors #1 and #2 are influenced by different velocity values. The total pressure comprises hydrodynamic pressure and hydrostatic pressure, as a lack of velocity distribution, the pressure distribution of the former is unclear while the latter always follows a triangular distribution. Therefore, the hydrodynamic pressure could be extracted from the total measured pressure with the known hydrostatic pressure distribution. That is, for each point along vertical direction, the measured hydrodynamic pressure (*MHd)* equals to the value of measured pressure (*Mp*) minus the hydrostatic pressure (*Hs*).

**Fig 7:**
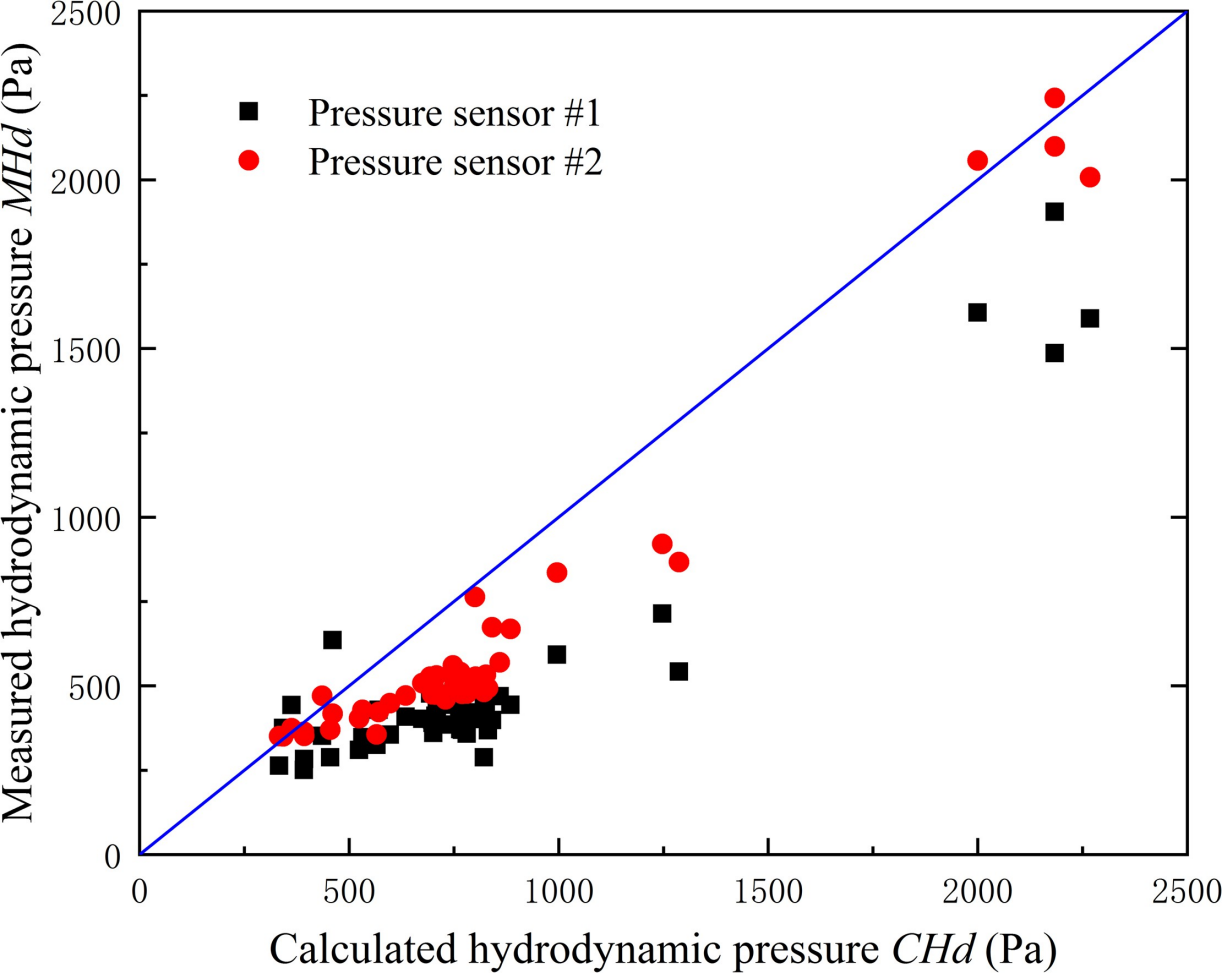
The comparison between measured hydrodynamic pressure (*MHd*) and calculated hydrodynamic pressure (*CHd*) of sensor #1 and sensor #2.

**Fig 8:**
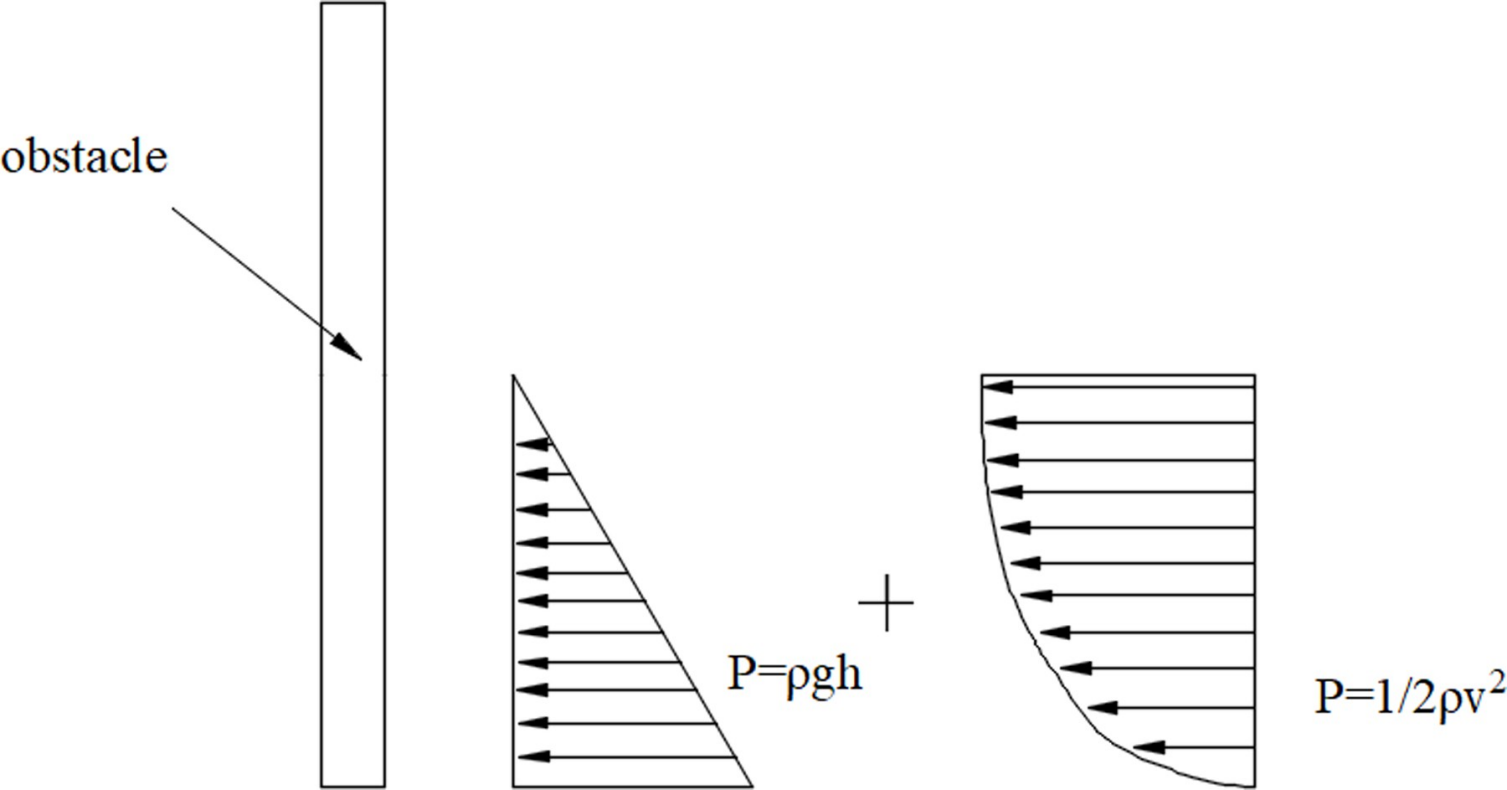
Schematic diagram of pressure distribution of fluid impact against an obstacle.

The comparison between measured hydrodynamic pressure (MHd) and calculated hydrodynamic pressure (*CHd*) by 1/2*ρV*^2^ is shown in Fig 7. It shows that *MHd* of sensor #2 is larger than that of sensor #1, as the two sensors were at different heights. What’s more, the scatter plots for both pressure sensors exhibit a similar trend following the 45° line. This means *MHd* of the two sensors follow a distribution of square of flow velocity. Thus, it is believed that the fluid impact pressure distribution comprises a hydrostatic pressure distribution and a square of flow velocity distribution (Fig 8).

### 3.3 The maximum pressure point against a passable obstacle

It is known that when an obstacle subjected to shear failure because of fluid impact, the damage point always lies in the location of the maximum pressure. As the fluid impact pressure distribution illustrated in Fig 8, the uppermost point experiences maximum hydrodynamic pressure and minimum hydrostatic pressure, while the bottom point experiences the opposite. The location of maximum pressure meets the condition as follow,

$$\frac{\partial \left(\alpha_{x}\frac{1}{2}\rho V^{2}+\rho gh\right)}{\partial h}=0$$ (12)

where *α*_*x*_ is an empirical coefficient.

To solve Eq (12) simply, it gives,

$$\frac{\partial \left(\alpha_{x}\frac{1}{2}\rho V^{2}\right)}{\partial h}=\frac{\partial (\rho gh)}{\partial h}=\rho g$$ (13)

Therefore, the location of the maximum pressure point could be determined with the distribution of the flow velocity remains known.

## 4 Conclusions

This paper studied the impact load of flash flood against a passable obstacle by flume experiment. Five different models for calculating the fluid impact pressure were compared and analyzed. Combining two existing models, a new model considering both hydrodynamic and hydrostatic pressure was proposed. In the new model, the pressure in low Froude number condition was calculated by hydrostatic pressure *ρgh* times a constant empirical coefficient, while in high Froude number condition it was counted by the hydrodynamic pressure 1/2*ρV*^2^ times another constant coefficient. It has showed good result and convenient to apply. Besides, the distribution of fluid impact was inferred to comprise a hydrostatic pressure distribution and a square of flow velocity distribution. Based on the form of the pressure distribution, the maximum pressure point was determined by making the gradient of square of velocity along depth equals to *ρg*.

## Supporting information

S1 Table Experimental data for the analyses. (XLSX)

S2 Table The relative error of calculated α_3_. (XLSX)

S3 Table The relative error of calculated α_4_. (XLSX)
